# Supplementary material for: From sequence to enzyme mechanism using multi-label machine learning
Source: BMC Bioinformatics. 2014 May 19;15:150. doi: 10.1186/1471-2105-15-150 (PMC4229970; doi:10.1186/1471-2105-15-150)
Supplement: Additional file 2 — Java code of ml2db. Additional file ml2db_code.tar.gz contains the Java source code to run the multi-label machine learning experiments and save the results to database. The code’s Javadoc is included. [file 1471-2105-15-150-S2.zip › additional file 2/ml2db/ml2db/doc/main/ml2db/weka/JaccardEuclideanDistance.html]

JaccardEuclideanDistance


JavaScript is disabled on your browser.


- Overview
- Package
- Class
- Use
- Tree
- Deprecated
- Index
- Help

- Prev Class
- Next Class

- Frames
- No Frames

- All Classes

- Summary:
- Nested |
- Field |
- Constr |
- Method

- Detail:
- Field |
- Constr |
- Method


main.ml2db.weka

## Class JaccardEuclideanDistance

- java.lang.Object
- - weka.core.NormalizableDistance
  - - main.ml2db.weka.JaccardDistance
    - - main.ml2db.weka.JaccardEuclideanDistance

- All Implemented Interfaces:
  :   java.io.Serializable, weka.core.DistanceFunction, weka.core.OptionHandler, weka.core.RevisionHandler

  ---

    

  ```
  public class JaccardEuclideanDistance
  extends JaccardDistance
  ```

  A Jaccard distance. If the Jaccard distance = 1 (maximum distance), the
  Euclidean distance + 1 is returned instead, to "disentangle" examples that
  are equal for the Jaccard distance.

  Version:
  :   22 Apr 2013

  Author:
  :   ldf

  See Also:
  :   Serialized Form

- - ### Field Summary

    - ### Fields inherited from class weka.core.NormalizableDistance

      `R_MAX, R_MIN, R_WIDTH`
  - ### Constructor Summary

    Constructors

    Constructor and Description

    `JaccardEuclideanDistance()`
  - ### Method Summary

    Methods

    | Modifier and Type | Method and Description |
    | --- | --- |
    | `double` | `distance(weka.core.Instance a, weka.core.Instance b)` |
    | `weka.core.EuclideanDistance` | `getEuclideanDistance()` |

    - ### Methods inherited from class main.ml2db.weka.JaccardDistance

      `distance, distance, getIntersection, getRevision, getUnion, globalInfo`
    - ### Methods inherited from class weka.core.NormalizableDistance

      `attributeIndicesTipText, distance, dontNormalizeTipText, getAttributeIndices, getDontNormalize, getInstances, getInvertSelection, getOptions, getRanges, initializeRanges, initializeRanges, initializeRanges, initializeRangesEmpty, inRanges, invertSelectionTipText, listOptions, postProcessDistances, rangesSet, setAttributeIndices, setDontNormalize, setInstances, setInvertSelection, setOptions, toString, update, updateRanges, updateRanges, updateRanges, updateRangesFirst`
    - ### Methods inherited from class java.lang.Object

      `equals, getClass, hashCode, notify, notifyAll, wait, wait, wait`

- - ### Constructor Detail


    - #### JaccardEuclideanDistance

      ```
      public JaccardEuclideanDistance()
      ```
  - ### Method Detail


    - #### distance

      ```
      public double distance(weka.core.Instance a,
                    weka.core.Instance b)
      ```

      **Specified by:**
      :   `distance` in interface `weka.core.DistanceFunction`

      **Overrides:**
      :   `distance` in class `JaccardDistance`


    - #### getEuclideanDistance

      ```
      public weka.core.EuclideanDistance getEuclideanDistance()
      ```


- Overview
- Package
- Class
- Use
- Tree
- Deprecated
- Index
- Help

- Prev Class
- Next Class

- Frames
- No Frames

- All Classes

- Summary:
- Nested |
- Field |
- Constr |
- Method

- Detail:
- Field |
- Constr |
- Method
